# Supplementary material for: Tunable quantum emitters on large-scale foundry silicon photonics
Source: Nat Commun. 2024 Jul 10;15:5781. doi: 10.1038/s41467-024-50208-0 (PMC11237125; doi:10.1038/s41467-024-50208-0)
Supplement: Supplementary file 1 — Supplementary Information [file 41467_2024_50208_MOESM1_ESM.pdf]

Supplementary Information for:
Tunable quantum emitters integrated on large-scale foundry silicon photonics

Larocque et al.

CONTENTS

Supplementary Note 1 - Fabrication Flow 2

Supplementary Note 2 - Apparatus for Cryogenic Testing 4

Supplementary Note 3 - Emitter Coupling 5

Supplementary Note 4 - Experimental chiplet to PIC coupling 7

Supplementary Note 5 - Transfer Accuracy 8

Supplementary Note 6 - Resonant pump rejection of the PIC 9

Supplementary Note 7 - Pulsed Laser Reflections 10

Supplementary Note 8 - Resonant Fluorescence Statistics 10

Supplementary Note 9 - Emitter properties under pulsed excitation 12

Supplementary Note 10 - Transmission Losses 12

Supplementary Note 11 - Photoluminescence spectrum repeatability 13

Supplementary Note 12 - Local Material Variations 13

Supplementary Note 13 - Nonvolatile Tuning 14

SUPPLEMENTARY NOTE 1 - FABRICATION FLOW

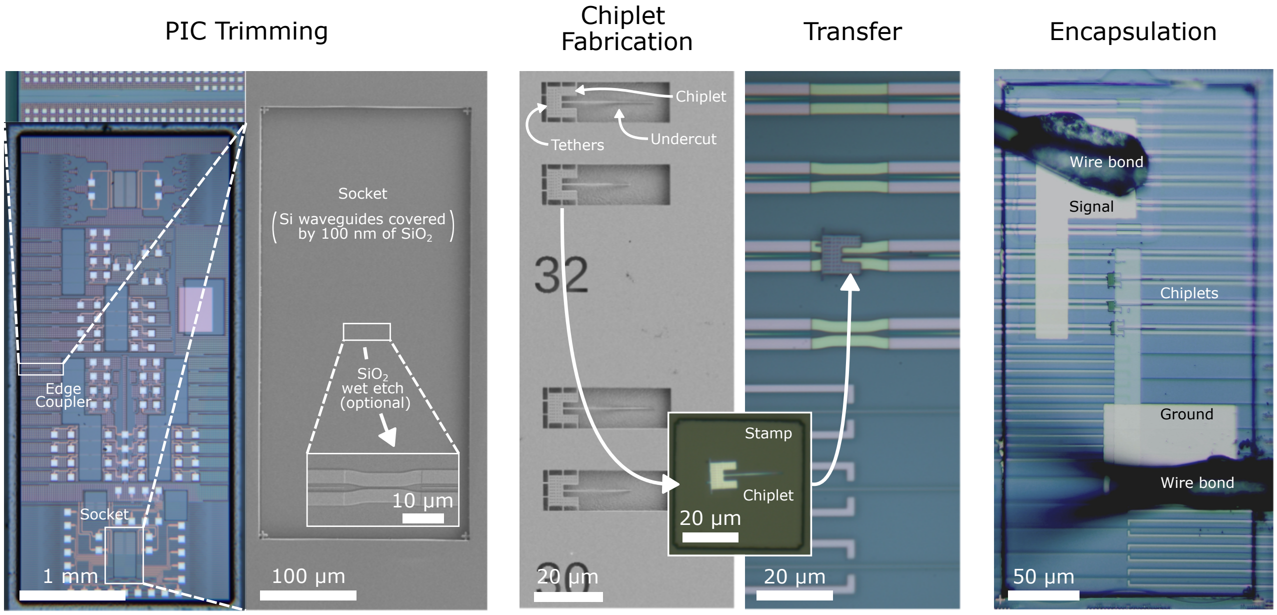

Supplementary Figure 1. **Hybrid photonic circuit assembly.** Images of the hybrid photonic integrated circuit (PIC) at various stages of its assembly along with some of the components used in the process.

To assemble our hybrid circuits, we opt for a transfer printing method [1], which has found success in integrating III-V materials onto photonic structures [2–7] and is now being industrially deployed for the assembly of micro-LED displays [8]. Fig. **1**

provides optical micrographs and scanning electron microscope images of the hybrid chip at various stages of the transferring procedure. The process flow starts with a silicon photonic integrated circuit (PIC) manufactured by a commercial foundry. We specifically obtained our chips from AIM photonics. The silicon waveguides used for this integration test are separated by 10 μm in order to avoid coupling between the edge couplers at the chip's facet, which expand optical modes to 4.1 μm diameters [9]. From coupled mode theory and finite element simulations, we expect less than 6×10^{-5}% of the light in a given silicon waveguide to couple into an adjacent one. Our chips also feature an etch stop layer laying 100 nm above the silicon layer. We use these etch blocks in the fabrication of openings in the > 5 μm PIC's silicon dioxide cladding, which we form with wet etching in a buffered oxide etchant. This 100 nm gap is a parameter set by the foundry and ensures a fixed distance between the silicon layer and the bottom of these trenches across the wafer. The inset in the "PIC Trimming" step of Supplementary Figure **1** shows how additional wet etching can further thin down this gap to the point of completely removing it and exposing the silicon layer of the chip.

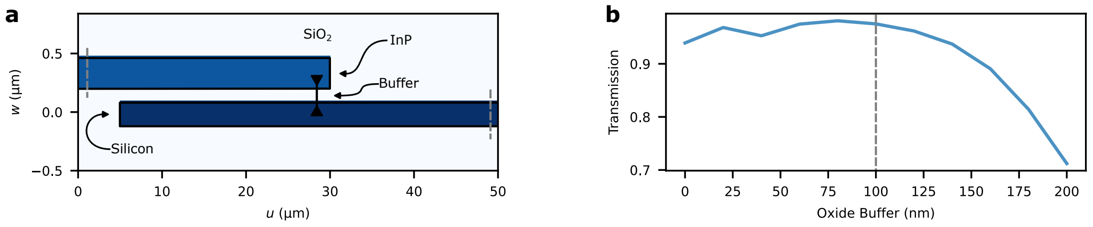

Supplementary Figure 2. **Finite difference time domain simulations (FDTD) of optical transmission from InP chiplets to silicon waveguides as a function of the gap separating them. a,** Schematics of the simulated system. The simulation launches the quasi TE mode of the InP waveguide and calculates its evolution via FDTD, where the InP guided mode adiabatically evolves into the quasi TE mode of the silicon waveguide. **b,** Overlap of the simulated field with the quasi TE mode of the silicon waveguide.

We calculate the influence of the thickness of this buffer on the optical transmission through this InP-silicon junction for the case of two perfectly aligned tapers via finite difference time domain (FDTD) simulations in Supplementary Figure **2**. Supplementary Figure **2**a shows the simulated structure. Given the high optical confinement of the waveguides, we consider a simulated window extending over $\Delta u = 50$ μm, $\Delta v = 1.6$ μm, and $\Delta w =$ buffer thickness + 1.5 μm surrounded by 200 nm perfectly matching layer boundaries. The corresponding grid size is (50, 20, 20) nm. As specified by our chiplet's design, the InP waveguide has a width of 410 nm, a height of 280 nm, and extends over 10 μm in the simulation before tapering down to a width of 100 nm over a length of 20 μm. As specified by our PIC design, the silicon waveguide has a width of 400 nm, a height of 220 nm, and extends over 5 μm in the simulation before tapering down to a width of 200 nm over a length of 40 μm. We place the tip of the silicon waveguide 5 μm from the start of $u = 0$. Supplementary Figure **2**b plots the overlap with the quasi TE mode at the output of the silicon waveguide of the simulated field at convergence with a tolerance of 10^{-5} resulting from launching the InP quasi TE mode into the chiplet. We consider a refractive index of 1.447 for the silicon dioxide, 3.195 for the InP, and 3.507 for the silicon along with an optical wavelength of 1300 nm. We observe > 90% transmission into the silicon waveguide up until a buffer thickness near 150 nm. To ensure a consistent buffer thickness between hybrid fabrication runs, we opted to maintain the default thickness established by the foundry's fabrication instead of further deepening the openings in the oxide cladding. Though such fabrication would have resulted in marginally more efficient transmission into the silicon PIC, it would have added uncertainty in the buffer thickness. Completely removing the cladding over the silicon would have been an alternative measure to ensure fabrication consistency. However, early fabrication runs that involved transferring the chiplets with a micro-probe [10] on exposed silicon waveguides such as the ones shown in Supplementary Figure **1**, had extremely low yields due to a lack of mechanical adhesion between the chiplets and the silicon PIC. Supplementary Figure **3** shows scanning electron microscope images of such trials trials. This lack of adhesion motivated the use of an oxide buffer layer, as transfer printing, which is a more scalable hybrid integration method [8] than probe-based "pick-and-place", requires stronger adhesion between the chiplets and PIC than between the chiplets and the transfer printing stamp that picked them up from the chiplet's native substrate. FDTD simulations also indicate marginally lower transmission efficiencies without a buffer, which likely stems from a reduction in the adiabatic nature of the mode converter.

To transfer the chiplets, we use a 50 μm × 50 μm polydimethylsiloxane (PDMS) stamp to pick up suspended InP nanophotonic devices with InAs quantum dots. [10–12]. These "chiplets" consist of an adiabatically tapered nanophotonic waveguide attached to a 10 μm ×10 μm pad. The contact between the pad and the stamp allows the chiplets to stick to the latter, thus breaking the tethers that originally held the devices in suspension as the stamp moves away from the surface. Due to its elastomer properties, contacting the stamp onto the silicon photonic circuit followed by gradually peeling it off leaves the chiplet on the circuit, thereby completing the transfer process. To ensure good alignment between the InP waveguide and the silicon photonic circuitry, we monitor the transfer procedure by placing the chip and the stamp with the chiplet underneath a microscope with a 0.41 numerical

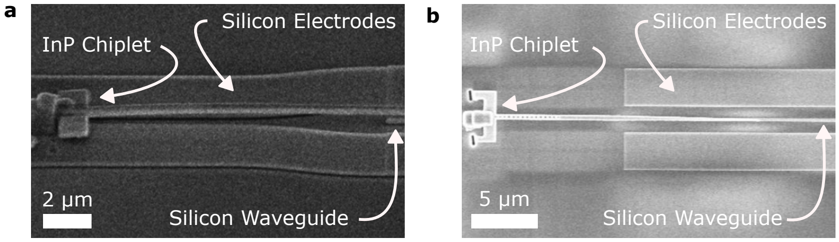

Supplementary Figure 3. **SEM images of InP chiplet transfer attempts with a micro-probe based transfer procedure. a,** Transfer on completely exposed silicon waveguides and electrodes. The lack of contact between the chiplet and the PIC caused by the topography resulting from the exposed silicon reduced the adhesion between the chiplet and the PIC, thereby exacerbating misalignment between the InP and silicon tapers during the transfer process. **b,** Transfer on partially exposed silicon waveguides and electrodes. Greater contact between the chiplet and the PIC in a part of the chip (left) with buried silicon electrodes improved the alignment between the InP and silicon tapers during the transfer process. The outline formed by the silicon electrodes in the left part of the image consist of a ghosting effect due to secondary electron scattering in the SEM image.

aperture (NA) objective with a long working distance.

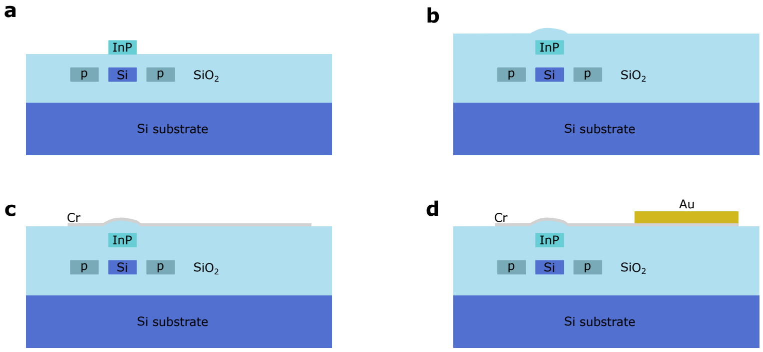

Supplementary Figure 4. **Encapsulation and wiring of the quantum dots. a,** Material stack at the start of the process following the transfer printing of InP chiplets embedding InAs quantum dots. Material labeled as p indicate p-doped silicon. **b,** PECVD deposition of 475 nm of silicon dioxide. **c,** Deposition of the ground plane covering the chiplets relying on electron-beam physical vapor deposition of a 20 nm layer of chromium patterned with photolithography and a liftoff process. **d,** Deposition of the electrical pads contacting the ground plane from **c** relying on electron-beam physical vapor deposition of a 50 nm layer of gold patterned with photolithography and a liftoff process.

Post-processing in a university cleanroom then allows the addition of further electronics to the hybrid chip. In our case, this additional circuitry consists of a ground plane covering the quantum dot chiplets with a silicon dioxide buffer layer between these two structures. Supplementary Figure **4** provides the process flow for completing the device. It starts off with a circuit with transfer printed InP chiplets (Supplementary Figure **4**a). We then add a 475 nm layer of silicon dioxide with plasma-enhanced chemical vapor deposition (PECVD) (Supplementary Figure **4**b). We then evaporate a 20 nm patterned layer of chromium (Supplementary Figure **4**c). This layer is thin enough to optically access the quantum dots from the top of the chip while also preserving sufficient conductivity for electronic purposes. We then complete the hybrid chip with the deposition of a 50 nm layer of gold providing pads for electrical probing (Supplementary Figure **4**d). Both the deposition of the patterned chromium and gold layers rely on the same liftoff process. Namely, we rely on direct writing photolithography to pattern a 1 μm layer of positive photoresist (S1813). We then deposit the metal with electron-beam physical vapor deposition. We complete the liftoff process by leaving the covered chip in N-Methyl-2-pyrrolidone (NMP) overnight followed by sonication.

SUPPLEMENTARY NOTE 2 - APPARATUS FOR CRYOGENIC TESTING

We package the hybrid chip by wire bonding it to a cryo-compatible printed circuit board (PCB) and load it into a 5 K cryostat (Montana Instruments) for testing. Supplementary Figure **5** provides an apparatus of the corresponding testing apparatus along with the schematics of the cryostat. The chamber includes a window placed over the surface of the chip allowing us to optically excite the quantum dots in the chiplets with a confocal microscope setup. We rely on several lasers to implement the excitation schemes involved in this work. A 780 nm tunable diode laser (Velocity TLB-6712) provides continuous wave above-band

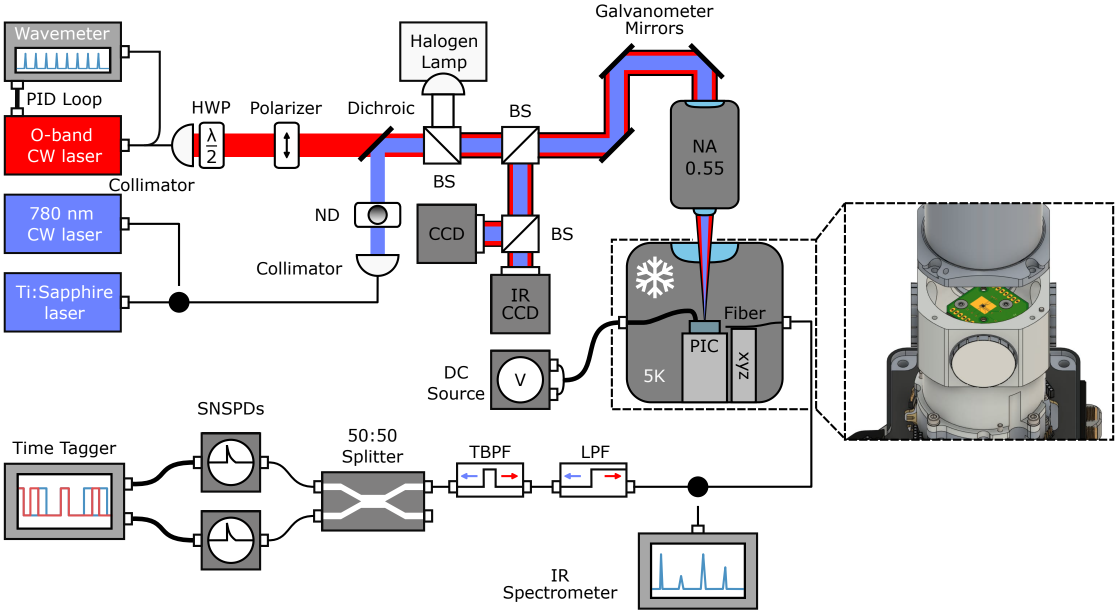

Supplementary Figure 5. **Apparatus for cryogenic testing.** Figure legend: CW: Continuous Wave, HWP: Half-wave plate, ND: Neutral density filter, BS: Beam splitter, IR: Infrared, CCD: Charge-coupled device camera, LPF: Long-pass filter, TBPF: Tunable band-pass filter, NA: Numerical Aperture, PIC: Photonic integrated circuit, xyz: xyz piezo-electrically actuated positioner, SNSPD: Superconducting nanowire single photon detector. Inset: schematics of our cryostat.

excitation whereas we use a Ti:Sapphire laser (Coherent MIRA-HP) for above-band pulsed excitation. For resonant and quasi-resonant excitation, we rely on an O-band tunable external cavity diode laser (Santec TSL-570) with additional polarization optics to control the polarization of the beam. We further stabilize the emission wavelength of this laser with an external PID loop based on the readout value of a wavemeter (HighFinesse WS-7) taping out a portion of the output laser light. Galvanometer mirrors can accurately change the imaging location on the chip for repeatable spatial control over the single-photon excitation location. Given the 0.55 NA of our imaging objective, we are able to resolve this excitation over a 0.92 μm spot-size. An external DC voltage source (Keithley 2400 Series) interfaces with the PCB through the cryostat's built-in electrical connections. A lensed fiber with a mode field diameter of 2.5 μm routed within the chamber with a sealed feedthrough collects single photons emitted by the quantum dots. To align the fiber with the PIC's waveguide, we place it on a piezo-electrically xyz positioner (Attocube). The fiber then interfaces with additional equipment such as a setup with removable optical filters before going towards our detection apparatus, which include a near-infrared spectrometer (Princeton Instruments Acton SP2500) and superconducting nanowire single photon detectors (SNSPDs) (Photon Spot). A streaming time-to-digital converter (Swabian Instruments Timer Tagger 20) converts detected counts from the detectors into digital data for correlation measurements. We use filters to remove the influence of parasitic photons for SNSPD measurements. A low-pass filter removes any above-band light that may be scattering into the fiber. A tunable-band pass filter blocks out the emission of other quantum dots that are residually excited by the above-band pump beams.

SUPPLEMENTARY NOTE 3 - EMITTER COUPLING

We attribute the varying degrees of counts from each emission line in Fig. 2b of the main text to different levels of coupling between the dipole radiation of the quantum dots and the waveguide, also know as the β factor of the emitters. We estimate this factor with MEEP FDTD [13] simulations modeling the quantum dot emitter as a Gaussian dipole source centered at 1300 nm with a 130 nm bandwidth. We rely on a spatial resolution of 20 nm and on the same geometric and material properties from the simulations in Supplementary Note 1. When the dipole is perfectly centered in the waveguide and its orientation is perpendicular to the waveguide, we obtain 84.4% coupling into its quasi TE0 mode. However, only half of this figure accounts for light that can couple to the silicon PIC as the other half would propagate in the opposite direction towards the InP chiplet's transfer printing pad. We examine how several factors could further affect this value. We first consider the relative orientation between the dipole and the waveguide for quantum dots with various displacements relative to the center of the waveguide. Supplementary Figure **6**a plots the resulting fraction of emitted power that goes towards the silicon PIC, indicating lower coupling within the

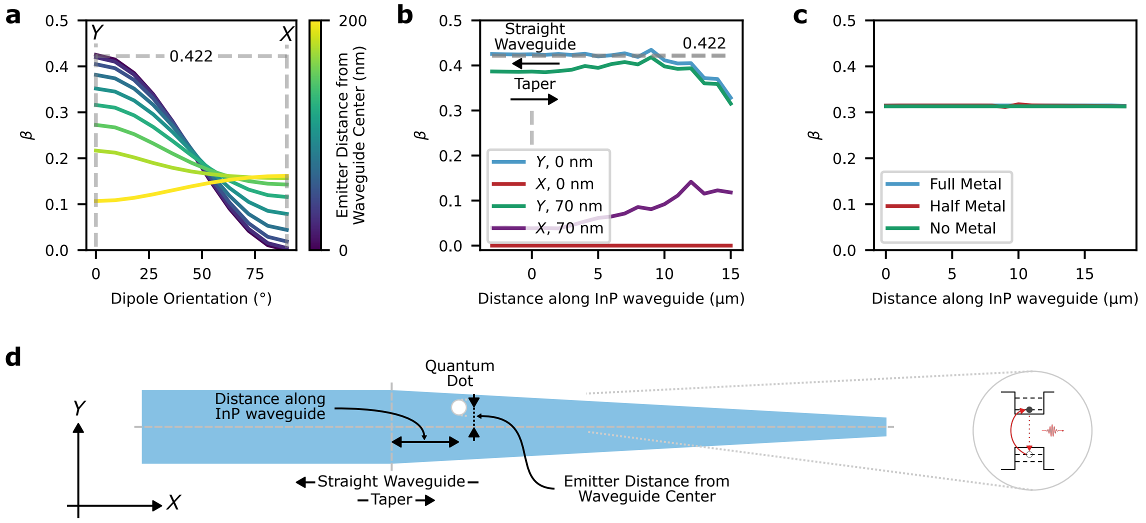

Supplementary Figure 6. Coupling efficiency, β factor, between a quantum dot emitter and an InP waveguide's quasi TE mode in the direction leading towards the InP-silicon junction. a, Coupling efficiency as a function of the relative angle between the waveguide and the dipole representing the quantum dot. Dashed lines label the orientations attributed to the sample's two dipoles, X and Y, when the orientation of the InP waveguide aligns with its crystal axis. We consider the optical coupling of quantum dots that are transversely displaced by up to 200 nm from the center of the waveguide. **b,** β factor as a function of the relative position of a quantum dot in a tapered InP waveguide. We provide the optical coupling of quantum dots that are transversely displaced by 0 nm and 70 nm from the center of the waveguide while considering both the contributions of the X and Y dipoles as defined in **a**. **c,** Influence of the ground electrode on the β factor of an emitter in a slab of InP. We consider simulations where the surface of the silicon dioxide layer covering the slab is entirely covered with metal, entirely free of metal, and where only positions with offset values below 10 µm are covered with metal, all featuring similar coupling factors. **d,** Schematic representation of the various parameters considered in the simulations shown in **a**-**c** that pertain to the geometry of the quantum dot relative to the InP waveguide.

quasi-TE mode when the dipole direction is parallel to the chiplet's waveguide or for quantum dots positioned away from the center of the waveguide. We also examine whether the position of the quantum dot along the taper affects its coupling to the waveguide. Supplementary Figure **6**b plots the coupled power directed towards one side of the waveguide for various positions of the quantum dot along the taper. We observe coupling similar to the case of a straight waveguide until the emitter is positioned farther than 10 µm down the taper length. In our case, this distance consists of half of the length of our taper. As shown in Fig. 2b of the main text, this work mostly considers emitters positioned before this point. Finally, we consider the influence of the chromium ground electrode placed over the InP chiplet, with material properties following a Drude-Lorentz model as parametrized in the MEEP materials library. The additional dispersion introduced by the metal in the simulations increases their duration to impractical lengths. For convenience, instead of considering coupling to a waveguide with a finite width, we choose to gauge the influence of this electrode by simulating coupling into a slab sharing the thickness of the waveguide, which allows us to run our simulations in a 2D geometry. We compare the results of such simulations in Supplementary Figure **6**c, where we consider both the presence and absence of the electrodes and do not observe any significant differences between both scenarios. We expect these results given that the 20 nm thickness of our chromium ground electrode is considerably lower than the material's skin depth, thereby preventing any significant forms of optical absorption. The results from Supplementary Figure **6** suggest that variations in the monitored counts amongst emitters likely stem from variations in the transverse position of the quantum dots along the InP chiplet relative to the center of the waveguide. For clarity, we provide schematics of these considered parameters relating the position of the quantum dot relative to the geometry of the InP waveguide in Supplementary Figure **6**d.

Besides emitter to waveguide coupling, other factors such as emission into the phonon sideband can limit the intrinsic efficiency of our single photon sources [14]. To estimate this factor, we fit the quasi-resonant emission spectrum of emitter E1 to the sum of a Voigt and a Gaussian distribution accounting for the zero-phonon line and the phonon sideband of the emitter, respectively. Supplementary Figure **7** shows the results of this fit, where the relative normalization factors indicate that 86 ± 1% of emission is in the zero-phonon line.

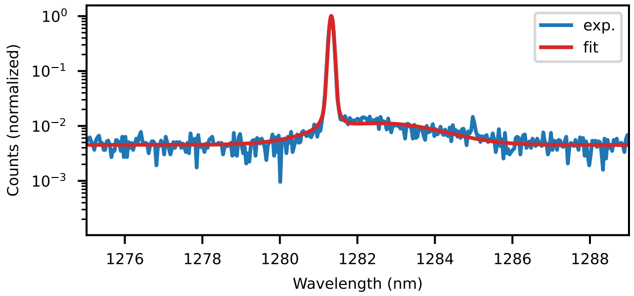

Supplementary Figure 7. **Emission spectrum of emitter E1 under quasi-resonant excitation.** Fitting the lineshape to the sum of a Voigt and Gaussian distribution suggests that 86 ± 1% of the quantum dot emission occurs in the zero-phonon line.

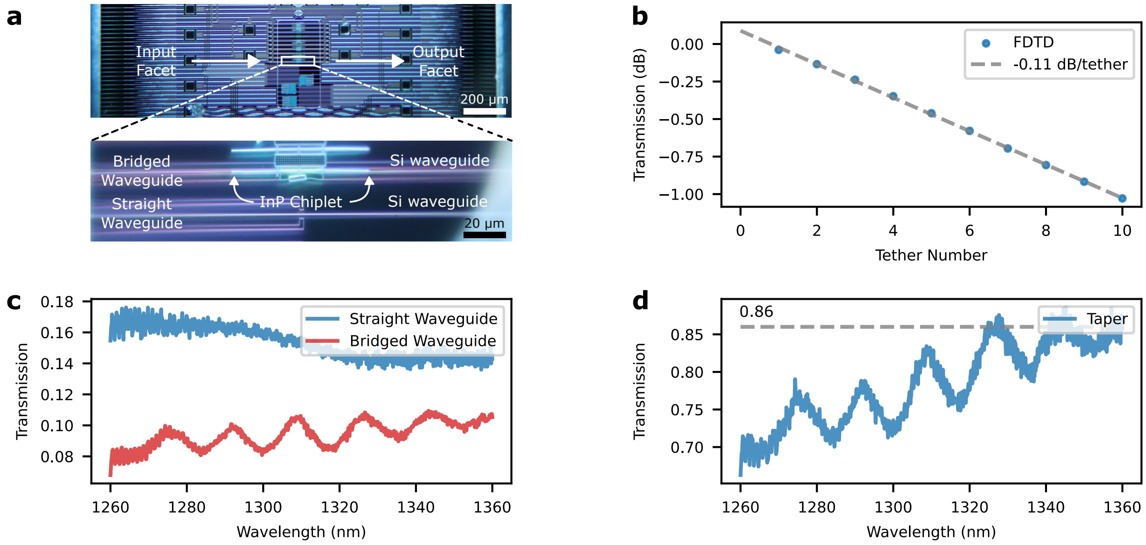

Supplementary Figure 8. **Chiplet to PIC coupling. a,** Dark field optical micrographs of the structure for gauging chiplet-to-PIC coupling effiency. An InP chiplet bridges a gap between two silicon adiabatic tapers to compare its transmission with a nearby straight silicon waveguide. Both the bridged and straight waveguides use the same edge couplers for fiber-to-waveguide facet coupling. **b,** Simulated transmission losses due to the tethers in the transferred InP chiplet shown in **a**. **c,** Transmission through the straight and bridged waveguides. **d,** Square root of the ratio of the curves shown in **c**, roughly corresponding to transmission at the taper junctions. The free-spectral range of the interference fringes roughly corresponds to that of a Fabry-Perot cavity sharing the length of the transferred InP chiplet.

SUPPLEMENTARY NOTE 4 - EXPERIMENTAL CHIPLET TO PIC COUPLING

To gauge the experimental feasibility of reaching ideal transmission values through the InP-silicon taper junctions of our hybrid structures, we test transmission through a straight waveguide with a gap between two tapered Si waveguides bridged with an InP chiplet. As shown in Supplementary Figure **8**a, the chiplet consists of a waveguide where both ends are tapered, thereby enabling incoming light to cross the silicon waveguide gap by means of optical coupling through the InP chiplet. Based on the procedure outlined in Supplementary Figure **9**, the relative offset and angle between the InP and silicon waveguides are 9 nm and -0.23°, respectively, which falls within the distribution shown in Supplementary Figure **11**. The chiplet is attached to transfer printing pads by means of 100 nm wide tethers where the waveguide width linearly expands from 550 nm to 650 nm and then back down to 550 nm over a distance of 2 μm. As in the case of the chiplet shown in Fig. 2 of the main text, the tapers are 20 μm long and have widths that linearly go down from 550 nm to 100 nm. Unlike the tuneable quantum dots, we do not deposit any silicon dioxide over this test structure, though a 100 nm silicon dioxide buffer still separates the chiplet from the silicon layer. The silicon tapers maintain their lengths of 40 μm and have a width that linearly go down from 400 nm to 200 nm.

We simulate the structure with the same FDTD parameters used in Supplementary Figure **2**. From these results, we calculate that transmission from the silicon taper to the InP chiplet is 98.7% whereas transmission from the chiplet to the silicon layer is

98.5%. As shown in Supplementary Figure **8**b, we also simulate transmission through various numbers of tethers supporting the waveguide, and extract transmission losses of -0.11 dB/tether. From these results, we estimate that transmission through the entire structure should be around 90% at best. Supplementary Figure **8**c plots the transmission spectrum of the bridged structure and of an adjacent straight waveguide. Both structures are 2 mm long and rely on edge couplers for fiber-to-waveguide facet transmission. We notice higher coupling efficiencies through the straight waveguide at lower wavelengths as opposed to the flat transmission over the O-band expected from our edge coupler's design [9]. We attribute this higher efficiency to our 400 nm silicon waveguides that are wider than the recommended 300 nm width, which likely better confines higher-order guided modes at lower wavelengths that would otherwise leak out of the waveguides after coupling into the chip. Supplementary Figure **8**d plots the square root of the ratio of the two curves from Supplementary Figure **8**c and provides an estimate of the transmission through each of the taper junctions of this test structure. We see that optical coupling through the chiplet is optimal within wavelengths of 1340 nm and 1360 nm, where we reach up to 86% coupling efficiency between the chiplet and the PIC. Removing the influence of the chiplet's tethers based on their simulated insertion loss increases this transmission metric to 90.2 %. Performing the same calculations at wavelengths of 1300 nm indicates an efficiency of around 79%, which roughly falls in line with the expected value from Fig. 2e of the main text.

SUPPLEMENTARY NOTE 5 - TRANSFER ACCURACY

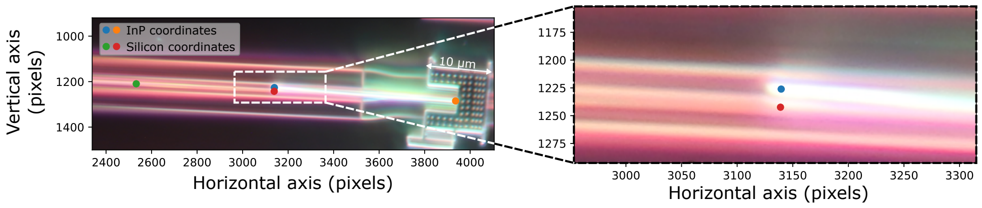

Supplementary Figure 9. **Extraction of alignment parameters.** We visually locate two points located on the InP (blue and orange) and silicon (red and green) waveguides. The blue point specifically lies on the end of the InP taper. We then convert the extracted pixel positions on the image to distance values based on a reference distance set by the size of the chiplets' transfer pad.

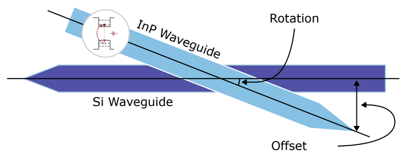

Supplementary Figure 10. **Alignment definition.** Sketch of misaligned InP and silicon tapers as defined in our hybrid architecture. The rotation misalignment parameter corresponds to the angle between the lines traced out by each waveguide. The offset parameter corresponds to the minimum distance between the tip of the InP waveguide and the line formed by the silicon waveguide.

We estimate the accuracy of our chiplet placement method by examining the relative alignment between 48 InP tapers and their underlying silicon waveguide on transferred chiplets distributed over 6 fabrication iterations of the chip shown in Supplementary Figure **1**. As illustrated in Supplementary Figure **9**, we visually locate two points on the integrated InP and native silicon waveguides of each hybrid device on optical micrographs collected with a NA=0.95 microscope objective. One of these points lies on the extremity of the InP taper. We then convert the resulting pixel values to absolute distance values. We perform this conversion with a factor extracted from 65 images of a known feature size on our device. Specifically, this distance corresponds to the width of the chiplet transfer pad, which is known to be 10 µm. We label this feature in Supplementary Figure **9** and found its corresponding pixel distance to be 274 ± 8 pixels, hence a conversion factor of 36 nm/pixel. Based on the standard deviation of this value, the error attributed to manually delimiting this length was 294 nm, which roughly corresponds to the diffraction limit of our microscope system.

Based on these positions, we extract the relative rotation and offset between the InP and Si waveguides. As shown in Supplementary Figure **10**, we define offset as the shortest distance from the Si waveguide to the tip of the InP taper. Supplementary Figure **11** provides a scatter plot of the extracted misalignment parameters. The figure features two sets of data. The first considers the tapers in all the chiplets that we transferred. The second set only considers tapers attached to chiplets that experienced an optimal transfer process: during our InP chiplet fabrication run, we observed an accumulation of residue in some parts of the

chip. This residue reduced the adhesion between the chiplets and the SOI PIC. Furthermore, some chiplets featured a preliminary tether design that left InP shrapnel on some parts of the chiplet, which also complicated the transfer process for some devices. To complete the transfer of such devices, we needed to considerably press the transfer printing stamp against the PIC while also introducing a substantial sheering motion to ensure that the chiplet would stick to the PIC as we peeled off the stamp. As seen in Supplementary Figure **11**, this sheering motion considerably misaligned the chiplets. The data also features strong correlations between the resulting offset and rotation parameters that directly trace back to the sheering motion that misaligned the chiplets. The mean and standard deviations of each data set, which follow their usual definitions provided in Eq. 1,

$$\mu_x = \frac{1}{n}\sum_{i=1}^{n} x_i, \qquad \sigma_x^2 = \frac{1}{n}\sum_{i=1}^{n}(x_i - \mu_x)^2, \tag{1}$$

are -3 ± 331 nm and $-0.018 \pm 0.685^{\circ}$ for the set formed by all the tapers and 13 ± 202 nm and $0.034 \pm 0.587^{\circ}$ for the set of those that had an optimal transfer. As mentioned earlier, this error is on par with the resolution of the optical imaging used in both the placement of the structures (NA=0.41) and the extraction of the resulting alignment parameters (NA=0.95), thus suggesting that our imaging systems are limiting our placement precision.

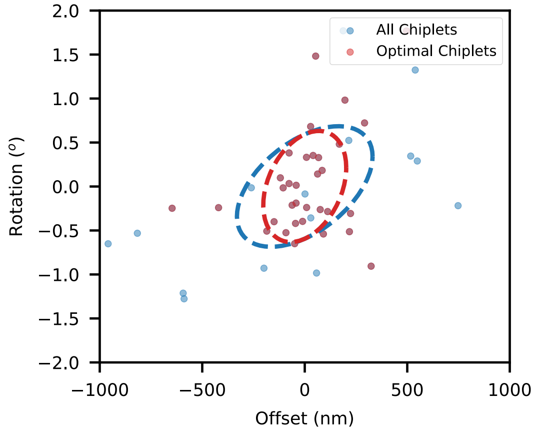

Supplementary Figure 11. **Placement accuracy.** Offset and rotation between the InP and Si waveguides of 48 InP-silicon taper junctions over 6 fabrication iterations of hybrid chips.

SUPPLEMENTARY NOTE 6 - RESONANT PUMP REJECTION OF THE PIC

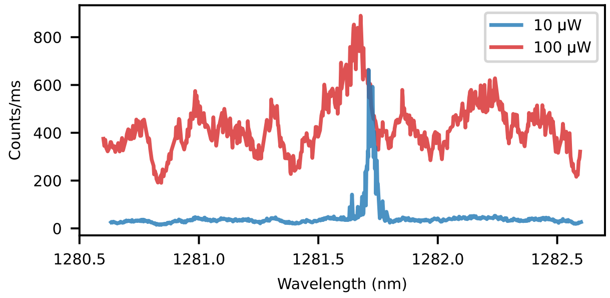

Supplementary Figure 12. Raw photoluminescence spectra while resonantly pumping the quantum dot with 10 μW and 100 μW of optical power.

We provide the raw photoluminescence excitation (PLE) data shown in Fig. 3aiii of the main text in Supplementary Figure **12**. Under the 10 μW pump power conditions reported in the main text, our waveguide features good pump rejection as indicated by the resonance fluorescence being roughly 45 times greater than the scattered laser signal. A tenfold increase in pump power drastically reduces this factor as the emitter reaches saturation and more laser power scatters into the PIC.

SUPPLEMENTARY NOTE 7 - PULSED LASER REFLECTIONS

We attribute the spurious peaks found in the pulsed autocorrelation measurement of Fig. 3b(i) of the main text directly to the output of our pulsed laser. We provide a correlation measurement between the optical output of the laser and an electrical trigger from a fast photodiode in Supplementary Figure **13**. In addition to the main pulses separated by roughly 13 ns, we observe secondary pulses that are most likely due to reflections.

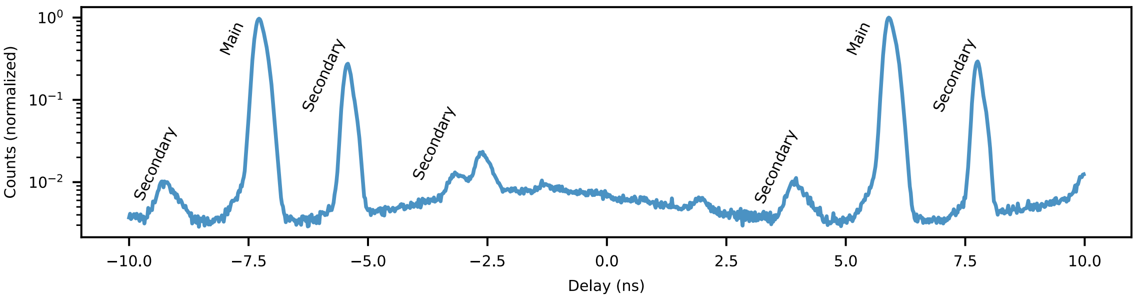

Supplementary Figure 13. **Pulsed laser output.** Autocorrelation measurement between the optical and electrical trigger outputs of the laser used in our pulsed excitation experiments.

SUPPLEMENTARY NOTE 8 - RESONANT FLUORESCENCE STATISTICS

To further investigate the source of the broad emission lines of our quantum dot's resonance fluorescence, we examine its photoluminescence excitation (PLE) spectra at various optical excitation powers. Supplementary Figure **14** shows the PLE of a quantum dot with excitation powers ranging from 0.08 μW to 243 μW. Lorentzian fits of the spectra indicate linewidths roughly ranging from 5 GHz to 11 GHz.

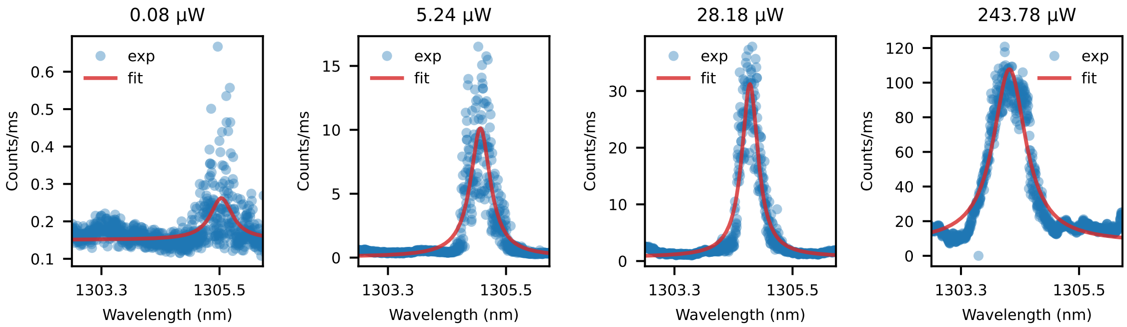

Supplementary Figure 14. Photoluminescence excitation spectra of the resonance fluorescence from a quantum dot emitter at various excitation powers. Lorentzian fits of the acquired spectra with laser powers of 0.08 μW, 5.24 μW, 28.18 μW, and 243.78 μW yield fitted linewidths of 8.0 ± 0.7 GHz, 7.2 ± 0.2 GHz, 5.8 ± 0.1 GHz, and 11.7 ± 0.2 GHz, respectively.

These broad linewidths at low excitation powers are consistent across various emitters in the chiplet. Supplementary Figure **15** shows the PLE spectrum of the resonance fluorescence from a total of four emitters at excitation powers of 5 μW. We observe linewidths roughly ranging from 4 GHz to 8 GHz, which have the same order of magnitude as the PLE linewidth of the quantum dot reported in Fig. 3 of the main text.

This consistent broadened linewidth suggests a common level of environmental fluctuations within the chiplet leading to decoherence effects. To estimate the magnitude of such perturbations, we fit the autocorrelation function of the resonance fluorescence reported in the main text to a model that accounts for dephasing and blinking. The fits reported in the main text relied on a double exponential function that usually describes a three-level model [15]

$$g^{(2)}(\tau) = 1 - (1 - A)\left((1 + B)e^{-|\tau-\tau_0|/\tau_1} - Be^{-|\tau-\tau_0|/\tau_2}\right), \tag{2}$$

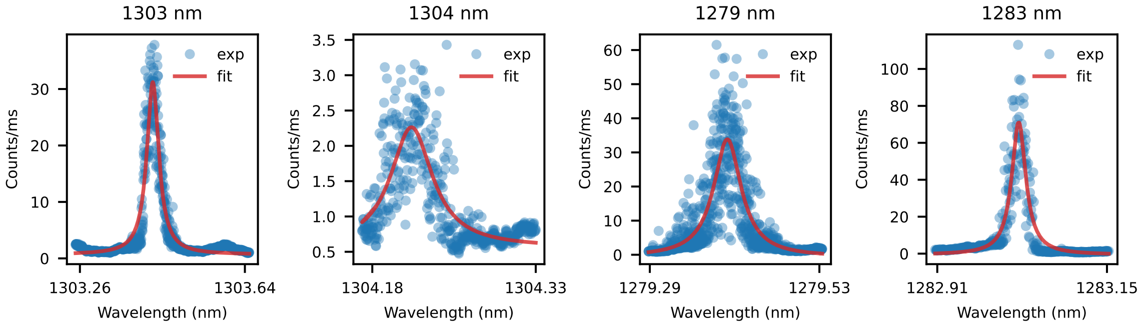

Supplementary Figure 15. Photoluminescence excitation spectra of the resonance fluorescence from four quantum dot emitters. Lorentzian fits of the acquired spectra for emitters with emission wavelenghts near 1303 nm, 1304 nm, 1279 nm, and 1283 nm yield fitted linewidths of 5.8 ± 0.1 GHz, 8.3 ± 0.5 GHz, 8.7 ± 0.4 GHz, and 4.4 ± 0.2 GHz, respectively.

convoluted with a Gaussian function with a width corresponding to the 170 ps jitter of our SNSPDs. As mentioned in the main text and shown in Supplementary Figure **16**, these fits suggest a $g^{(2)}_{\mathrm{Fit}}(0) = 0.08 \pm 0.03$ for an infinitely fast detector. Laser background from our excitation laser is likely responsible for this relatively large value. The contribution of background to the $g^{(2)}(0)$ value is $2\xi - \xi^2$, where $\xi = \mathrm{counts}_{\mathrm{background}}/\mathrm{counts}_{\mathrm{RF}}$ [16]. Based on our resonance fluorescence spectrum from Supplementary Figure **12**, we estimate $\xi = 0.056$, hence a contribution of around 0.11, which is similar to our fitted $g^2(0)$. To directly account for dephasing effects, we replace the above by [17]

$$g^{(2)}(\tau) = \left(1 - Ae^{-2\pi\eta\tilde{\tau}}\left(\cos(2\pi\mu\tilde{\tau}) + \frac{\eta}{\mu}\sin(2\pi\mu\tilde{\tau})\right)\right)\left(1 + Be^{-2\pi\Gamma_b\tilde{\tau}}\right), \tag{3a}$$

$$\eta = \frac{3\Gamma + 2\Gamma_d}{4,} \tag{3b}$$

$$\mu = \sqrt{\left(\frac{\Omega}{2\pi}\right)^2 - \left(\frac{\Gamma - 2\Gamma_d}{4}\right)^2}, \tag{3c}$$

$$\tilde{\tau} = |\tau - \tau_0|, \tag{3d}$$

where Γ is the lifetime-limited linewidth, Γ_d is the dephasing rate, and Ω is the Rabi frequency. We also introduce a term that accounts for blinking with amplitude B and rate Γ_b. Supplementary Figure **16** shows the fit of the resonance fluorescence data from Fig. 3c of the main text to Supplementary Equation (3) convolved with the 170 ps response of our SNSPDs. Here, we set $\Gamma = 126$ MHz based on the lifetime-limited linewidth reported in the main text and assume $\Omega \ll \Gamma$. From this fit, we obtain a dephasing rate of 1.2 ± 0.7 GHz. We primarily attribute these dephasing processes to pure dephasing and spectral diffusion, assuming that these occur within the lifetime of the emitter. To further disambiguate the influence of these two processes on resonance fluorescence, which are potentially characterized by long (charge noise) and short (phonon-induced dephasing) timescales, we estimate the pure dephasing rates of our quantum dots based on their expected Hong-Ou-Mandel interference visibility. We rely on the value reported in [12], given that these dots were grown on the same wafer as ours. For quantum dot emitters, this visibility V relates to to Γ_d and Γ as follows [18]

$$V = \frac{\Gamma}{\Gamma + 2\Gamma_d}. \tag{4}$$

Therefore, for the $V = 0.2$ value reported in [12], we estimate a pure dephasing rate of $\Gamma_d = 252$ MHz in our quantum dot sample. With this value, we consider a numerical model of the second-order correlation function of the field scattered by a two-level system after resonant interaction with a continuous-wave laser, described by the following time-independent Hamiltonian:

$$\hat{H}_{\mathrm{2lvl}} = \Delta\hat{\sigma}_-\hat{\sigma}_+ + \frac{\Omega}{2}(\hat{\sigma}_- + \hat{\sigma}_+), \tag{5}$$

where we introduce the raising and lowering operators $\hat{\sigma}_+$, $\hat{\sigma}_-$, and Δ as the frequency detuning of our pump laser relative to the emission frequency of the quantum dot. We then fit our autocorrelation data to a weighted average of the model over Δ:

$$g^{(2)}(\tau) = \int_{-\infty}^{+\infty} \frac{1}{\sigma_{\mathrm{SB}}\sqrt{2\pi}} e^{-\frac{1\Delta^2}{2\sigma_{\mathrm{SB}}^2}} g^{(2)}_{\mathrm{2lvl}}(\tau, \Delta)\, d\Delta, \tag{6}$$

where we introduce σ_{SB} as the broadening attributed to spectral diffusion. From our fits shown in Supplementary Figure **16**, we extract $\sigma_{\mathrm{SB}} = 389.2 \pm 0.2$ MHz.

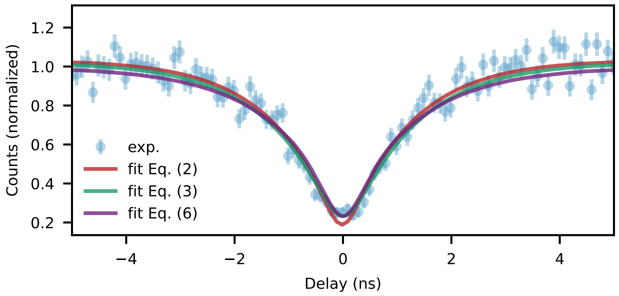

Supplementary Figure 16. **Second-order autocorrelation data fits.** Resonance fluorescence autocorrelation data from our hybrid integrated quantum dot emitters fitted to various models. Error bars on experimental data, exp., correspond to the Poisson noise, $\sqrt{N}$, attributed to the detected counts, N. We consider the three-level emission fit from the main text and Supplementary Equation (2) convolved with our detector's 170 ps response. We also fit our data to a model of resonance fluorescence from Supplementary Equation (3), which accounts for blinking and dephasing that doesn't distinguish between pure dephasing and spectral diffusion. Finally, we consider the model of resonance fluorescence from Supplementary Equation (6) that accounts for pure dephasing and introduces a detuning between the laser and emitter frequencies to incorporate spectral diffusion.

SUPPLEMENTARY NOTE 9 - EMITTER PROPERTIES UNDER PULSED EXCITATION

Here, we report the saturation powers and lifetimes of other emitters under above-band excitation using a 776 nm pulsed laser with a pulse width of 120 fs and a 80 MHz repetition rate. Supplementary Figure **17** provides the saturation curve for five different emitters, E1-E5, whereas Supplementary Figure **18** plots their corresponding radiative decay time traces. We observe that the saturation power, P_{sat}, of our emitters ranges from 0.14 µW to 0.57 µW, whereas the dominant lifetime, τ, varies within 0.8 ns to 2.58 ns. The influence of a second time constant, $\tilde{\tau}$, which ranges from 6.9 ns to 14.7 ns, is more noticeable in emitters with shorter lifetimes, e.g. E1 and E4, thereby suggesting the presence of an adverse carrier recombination process to radiative recombination in the quantum dots.

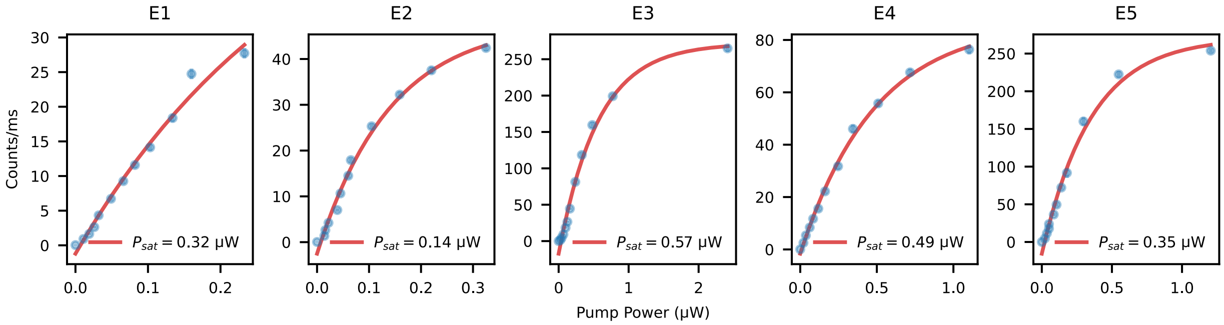

Supplementary Figure 17. **Saturation measurements of emitters along the InP waveguide.** Saturation curves of five emitters (E1-E5) along a transferred chiplet waveguide under pulsed above-band excitation at a wavelength of 776 nm using a NA=0.55 objective. We also provide fits of the data to $R(P) = R_{\text{sat}}(1 - \exp(-P/P_{\text{E1,sat}}))$ and include the fitted P_{sat} in each emitter's subpanel.

SUPPLEMENTARY NOTE 10 - TRANSMISSION LOSSES

To estimate the efficiency of our hybrid chip as a single-photon source, we compare the counts monitored on the SNSPDs during saturation measurements under pulsed above-band excitation shown in Supplementary Figure **17** to the 80 MHz repetition rate of the exciting laser. Emitters 3 and 5 yielded the largest count rates with values near 250 kHz, thereby suggesting an overall system efficiency of around 0.3%. We attribute a portion of this value to the insertion loss of the bulk and fiber optical components placed between the detectors and the lensed fiber collecting single photons from the hybrid chip. We provide the transmission of each component in Supplementary Table **1**, which amount to a total of 2.4% when considering the average transmission of the 50:50 splitter.

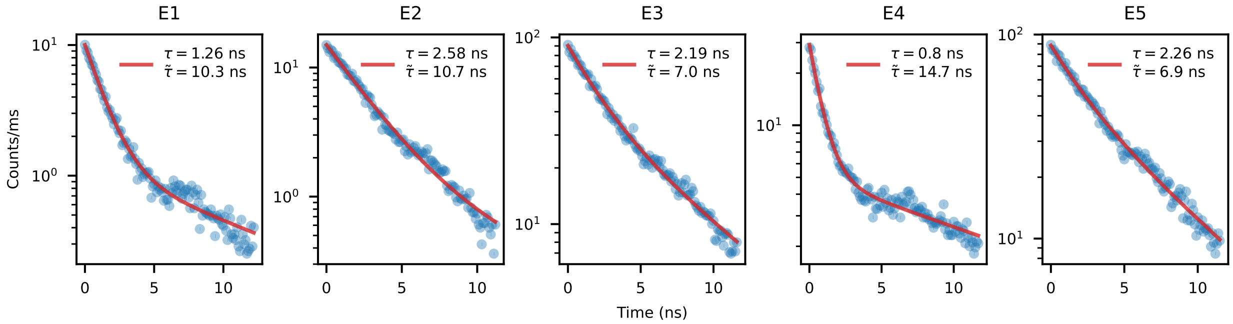

Supplementary Figure 18. **Lifetime measurements of emitters along the InP waveguide.** Radiative decay time trace of five emitters (E1-E5) along a transferred chiplet waveguide. We also provide fits of the data to a double-exponential decay function $R(t) = a\exp(-t/\tau) + \tilde{a}\exp(-t/\tilde{\tau})$, where $a > \tilde{a}$. We include the best fit values for τ and $\tilde{\tau}$ of each emitter in their respective subpanels. We collected the data with the same equipment used in Supplementary Figure **17** and a third of the maximum pump power considered in the corresponding saturation measurement.

Optical	Transmission
Tunable bandpass filter	0.61
Long pass filter	0.68
Optical fiber leading to SNSPDs	0.55
50:50 Splitter – Port 1	0.5
50:50 Splitter – Port 2	0.45
SNSPD Detection Efficiency	0.22

Supplementary Table 1. **Optical component transmission losses from bulk and fiber optics.** Transmission losses of the optical components placed between the SNSPDs and the lensed fiber collecting single photons from the hybrid chips.

We can also account for losses attributed to our hybrid PIC components. Though we measured > 50% coupling efficiency from the facet of the chip into a lensed fiber with a 5 μm mode field diameter, the optical feed-through leading into the cryostat relied on a 2.5 μm mode field diameter fiber, which reduced the coupling efficiency to 38%. Additional experiments comparing a chip provided directly by the manufacturing foundry to one that undergoes the hybrid assembly flow from Supplementary Figure **1** indicate that our post-processing flow, which relies on wet etches and lift-off processes, can reduce coupling efficiency at the facet to 80% of its original value. Furthermore, misalignment between the InP chiplet's and the silicon PIC's adiabatic tapers induce further loss. By extracting the alignment between the two tapers using the methodology from Supplementary Note 5, we estimate a relative waveguide offset of 20 ± 2 nm and angle of 0.44 ± 0.05° between the InP and silicon tapers. Based on the simulations from Fig. 2e of the main text, we expect 70% transmission through this structure. These losses amount to roughly 21.28% transmission from the chiplet to the fiber.

Combining the losses from bulk and fiber optics, integrated PIC components, and emitter coupling to the chiplet gives us a system efficiency estimate of 0.26%. This is slightly below our measured value of 0.3%, which we attribute to overestimates in the losses at the chip's facets and at the InP-silicon adiabatic taper junction.

SUPPLEMENTARY NOTE 11 - PHOTOLUMINESCENCE SPECTRUM REPEATABILITY

Supplementary Figure **19**, plots the extracted linewidth and brightness of emitter E2 discussed in the main text. We extract these quantities by fitting the emission spectra of the emitter under above-band excitation with a CW laser with a wavelength of 780 nm acquired while we sweep the voltage from -200 V to 200 V across our tuning device. Figure 4a from the main text displays some of these spectra. We observe that the extracted quantities from two sweeps are very similar for a given voltage. We attribute minor discrepancies to factors such as drift in the position of our excitation beam and the finite resolution of our specrometer, which is around 7 GHz.

SUPPLEMENTARY NOTE 12 - LOCAL MATERIAL VARIATIONS

The trends in linewidth broadening and dimming exhibited by emitter E2 shown in Fig. 4a of the main text are not universal to all emitters. Though E2 experiences these phenomena at low voltages, i.e. a large reverse bias as displayed in Supplementary

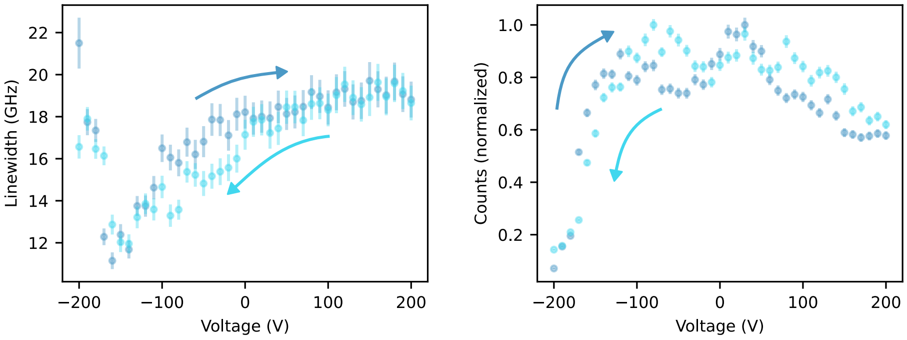

Supplementary Figure 19. **Linewidth and brightness of emitter E2 with applied voltage.** The values are extracted from the optimal parameters for fits of measured spectra to Lorentzian lineshapes. Error bars correspond to one standard deviation of the fitted parameter (see Methods). We provide data taken over two voltage scans. The direction of the arrows included in the plots provide the direction of the voltage scan for its correspondingly colored data set.

Figure **20**a, others can experience them at large biases in general or only at a large forward bias, as shown in Supplementary Figures **20**b,c, respectively.

Prior work reports similar observations. Though local strain can have significant influence on the emission properties of quantum dots [19], it can also influence their response to Stark tuning [20, 21]. Inhomogeneous stress imparted by our PECVD oxide buffer onto our transferred chiplets could justify similar behavior observed in our own samples.

SUPPLEMENTARY NOTE 13 - NONVOLATILE TUNING

Spectrally tuning individual quantum dots within a single InP chiplet would require complex control electronics while also placing extreme demands on the tuning electrode placed beneath it in terms of spatial resolution and wiring. Here, we investigate using the carrier redistribution effects observed in Fig. 4 of the main text for an entirely different approach to Stark shift control at individual quantum dot locations: gate-controlled charge trapping of photogenerated carriers. Modeled by surface charges of $+\sigma$ ($-\sigma$) on the top (bottom) of the InP waveguide, these act as non-volatile floating gates that modulate the electric field across the quantum dot layer. We illustrate this effect in the inset of Fig. 1a of the main text.

The quantum confined Stark effect [11, 22, 23] accounts for the frequency shift resulting from the perpendicular electric field applied across the dot, F_w:

$$h\Delta\nu = pF_w + \beta F_w^2 + O(F_w^3), \tag{7}$$

$$F_w = \alpha(\Delta V/d) + F_0, \tag{8}$$

$$p/h = a(d/\alpha) - 2b(d/\alpha)^2 F_0, \tag{9}$$

$$\beta/h = b(d/\alpha)^2, \tag{10}$$

where p and β account for the dipole moment and the polarization of the dot, respectively. This quadratic dependence lines up with the emission frequency shifts that we observed in Fig. 4b of the main text. Here, we introduce $d = 2.44$ µm as a characteristic electrode spacing weighted by the fringing in the electric field as seen in Fig. 1a of the main text; the free variables α and F_0 capture the reduction and offset of the electric field at the quantum dot due to free carriers, respectively. Fitting this model with separately measured values of p and β [11] indicates that the strong electric field screening under above-band excitation ($\alpha_{\text{ab}} = 0.017$) is improved under quasi-resonant excitation ($\alpha_{\text{qr}} = 0.033$). Both conditions entail a similar offset field of $F_{0,\text{ab}} = 59$ kV/cm and $F_{0,\text{qr}} = 60$ kV/cm, respectively.

To examine the role of charge distribution on the proposed screening, we proceed by applying a sequence of ΔV to the device under different illumination conditions. Supplementary Figure **21** depicts the steps involved in the sequence following steps of the sequence: write – illuminating the chiplet with above-band light while ramping from $\Delta V = 0$ to a "write" value $\Delta V = V_w$; read – probing $\nu_{\text{qr}}(\Delta V = 0)$ under quasi-resonant excitation. Figure 4d of the main text plots this write-read sequence for emitter E2 as labeled in Fig. 2b of the main text. Starting from $(\Delta V, \nu_2 - \nu_2(0)) = (0\text{ V}, 15\text{ GHz})$ probed under quasi-resonant excitation, program the dot to $(0\text{ V}, 15\text{ GHz}) \rightarrow (-200\text{ V}, 22\text{ GHz}) \rightarrow (0\text{ V}, -16\text{ GHz})$. Likewise, programming the dot to (200 V,-40 GHz) under above-band illumination reverts the state of the dot back to (0 V, 15 GHz). This shows the ability to write ν_2 so that quasi-resonant excitation produces read values within 30 GHz. We attribute this memory effect to non-volatile charge redistribution

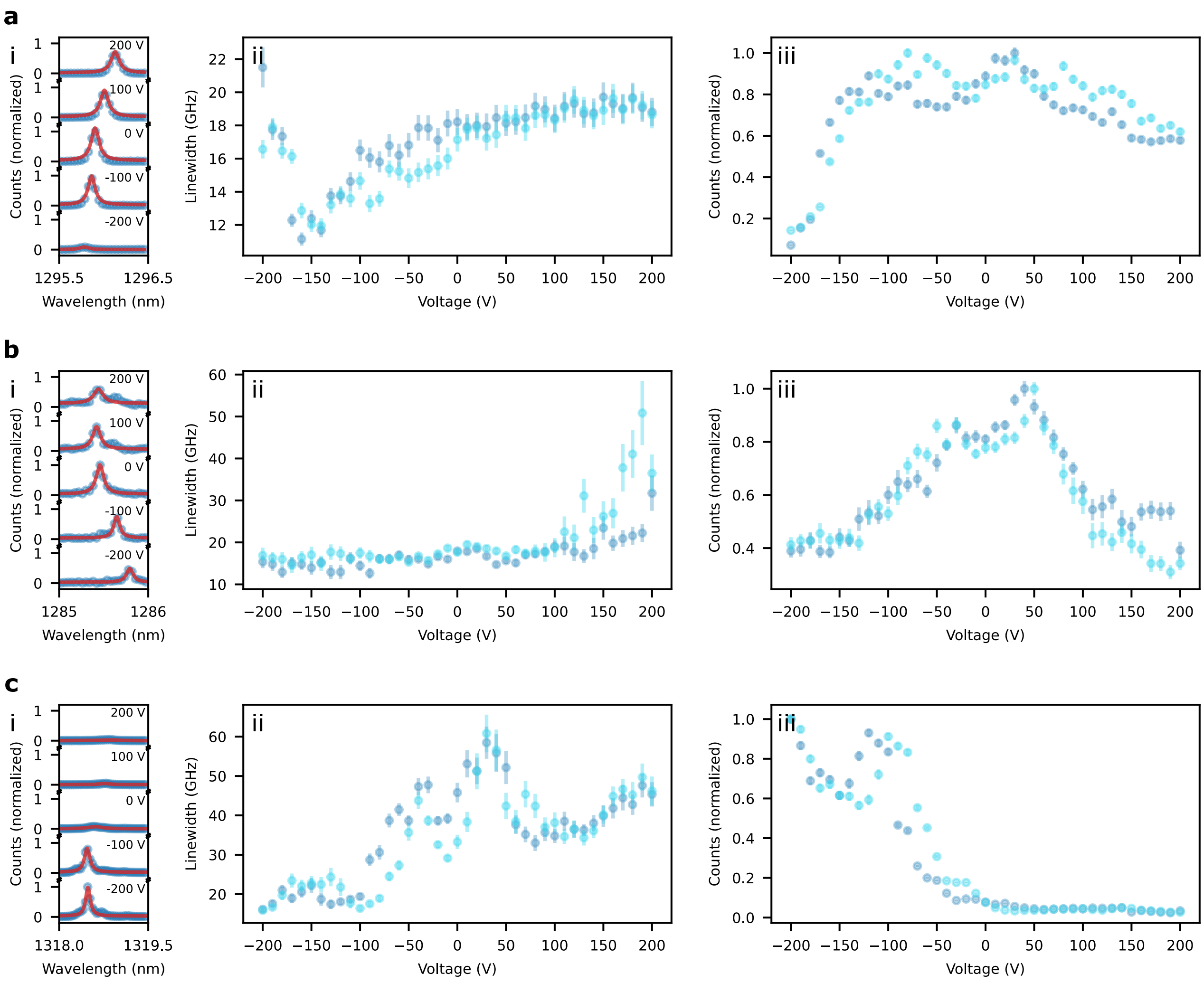

Supplementary Figure 20. **Local material variations.** Applied voltage dependence of the emission properties of three emitters featuring dimming at **a,** low, **b,** low and high, and **c,** high applied voltages. The properties illustrated here include i, the emission line of the emitter at five different voltages, ii, the applied voltage dependence of the emitter linewidth, and iii, the applied voltage dependence of the emitter's relative brightness. The linewidth and relative brightness values correspond to the optimal parameters for fits of measured spectra to Lorentzian lineshapes. Error bars correspond to one standard deviation of the fitted parameter (see Methods). Data sets attributed to different voltage sweeps are correspondingly colored.

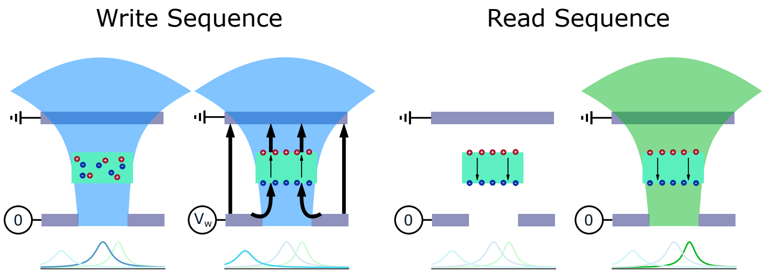

Supplementary Figure 21. **Illumination sequence for carrier redistribution.** Sequence taken to alter the internal electric field in the III-V chiplet. Above-band illumination first excites carriers in the chiplet. Applying a voltage to the device then redistributes the carriers in the device. Turning off the illumination and the voltage subsequently immobilizes the charges which apply an internal field to the quantum dots. Emission variations under quasi-resonant excitation in turn provide information regarding this internal field.

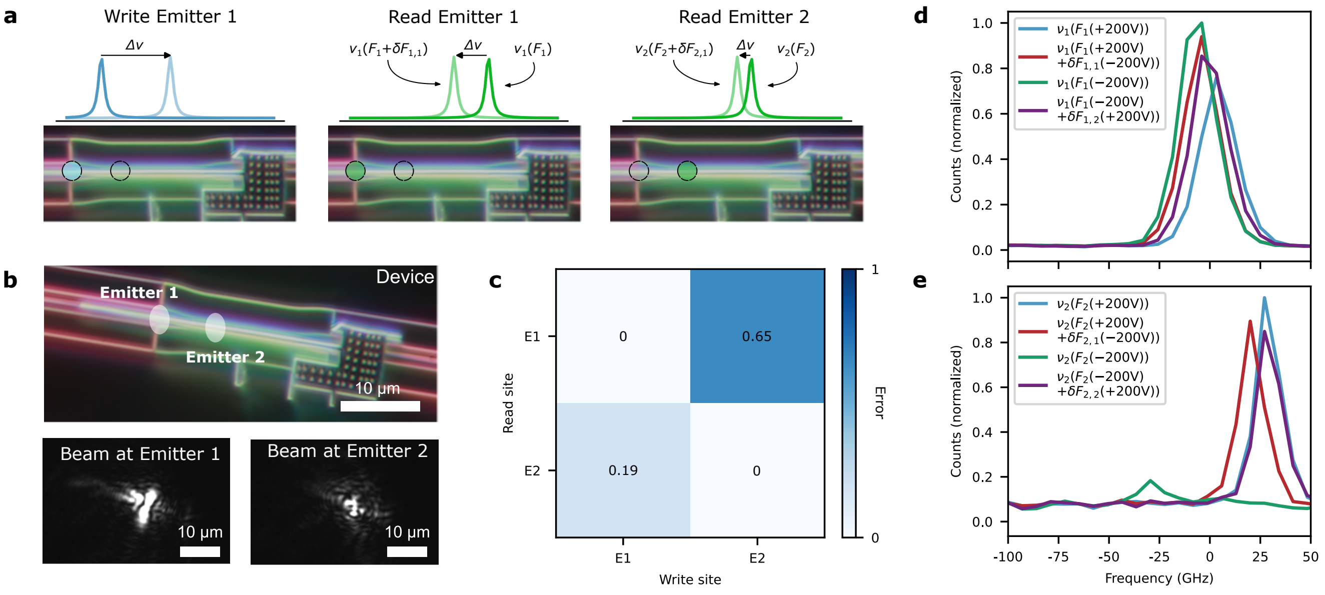

Supplementary Figure 22. **Emission frequency tuning with carrier redistribution a,** Sketch of the carrier redistribution tuning scheme for the illustrative case where $i = 1$ and $j = 2$. Above-band light illuminates emitter E1 while setting the bias across the tuning electrodes to $\Delta V \neq 0$. We then turn off the above-band light followed by the bias and read out the emission frequency, $\nu_1(F_1 + \delta F_{1,1})$, quasi-resonantly to estimate the change in the ambient electric field applied on emitter E1, $\delta F_{1,1}$, due to carrier redistribution around emitter E1. We then quasi-resonantly measure the emission frequency of emitter E2, $\nu_2(F_2 + \delta F_{2,1})$, to probe frequency shifts caused by a change in the electric field applied on emitter E2, $\delta F_{2,1}$, arising from redistributing carriers around emitter E1. We label frequency shifts arising from these various sequences of applied voltage biases and illuminations as $\Delta\nu$. **b,** Location of emitters E1 and E2 along the chiplet in addition to the profile of the focused above-band laser beam while redistributing carriers around them. **c,** Residual normalized electric field experienced by a quantum emitter while tuning the electric field of another. **d,** Quasi-resonant emission spectra of emitter E1 and **e,** emitter E2 used to obtain the matrix shown in **c**.

near the quantum dot.

To characterize the crosstalk of this non-volatile memory, we perform an illumination sequence from Supplementary Figure **21** on one dot, e.g. dot i, followed by a measurement of the emission wavelength under quasi-resonant excitation. We then run an additional quasi-resonant measurement on a dot located ~ 5 µm away from the first one, e.g. dot j. This procedure allows us to measure how changing the internal field applied to the one dot influences the internal field on the other. Specifically, for dot j, we obtain the frequency shift $\Delta\nu_j$ by letting $F_0 \rightarrow F_j + \delta F_{j,i}$ in Eq. (7), i.e.,

$$h\Delta\nu_j = p_j(F_j + \delta F_{j,i}) + \beta_j(F_j + \delta F_{j,i})^2, \tag{11}$$

where F_j is the initial internal field on dot j and $\delta F_{j,i}$ is the modification of this field following the above-band illumination of dot i. We illustrate this process for emitters E1 and E2 in Supplementary Figure **22**a, where we draw out the corresponding excitation locations in the chiplet and expected shifts in emission for each emitter. Partial exposure to the optical field illuminating dot i may cause a crosstalk on dot j, i.e., $\delta F_{j,i} \neq 0$. In practice, an aberrated illumination beam may cause such crosstalk. As shown in Supplementary Figure **22**b, the distorted profiles of the laser beam while exciting the emitters strongly suggests the presence of such aberrations in our system. We attribute them to bending and deformations in our cryostat's window that we use to optically excite the emitters with the confocal microscope shown in Supplementary Figure **5**. Even if completely mitigated, the diffraction limit will still lower-bound $\delta F_{j,i}$. We quantify the error from this crosstalk by $|(\delta_{j,i}\,\delta F_{j,j} - \delta F_{j,i})/\delta F_{j,j}|$, where $\delta_{j,i}$ is the Kronecker δ function. As seen from the crosstalk matrix in Supplementary Figure **22**c, we observe a maximum crosstalk of 0.65 in the off-diagonals for emitters separated by roughly 5 µm.

Supplementary Figures **22**d,e provide the spectra from emitters E1 and E2, respectively, used to extract these crosstalk values. We acquired the plotted data by first flooding the chiplet with above band light while biased at $\Delta V = +200$ V. We then quasi-resonantly read out emitters E1 and E2, which give us $\nu_1(F_1(+200\text{V}))$ and $\nu_2(F_2(+200\text{V}))$. As drawn in Supplementary Figure **22**a, we expose emitter E1 to above-band light with roughly 1.5 µW, which is five times greater than the fitted saturation value based on Supplementary Figure **17**, while biased at -200 V. After turning off the laser and the bias, we quasi-resonantly measure the emission of emitters E1 and E2, which gives us the $\nu_1(F_1(+200\text{V}) + \delta F_{1,1}(-200\text{V}))$ and $\nu_2(F_2(+200\text{V}) + \delta F_{2,1}(-200V))$ spectra. We then flood the chiplet with above band light while biased at $\Delta V = -200$ V. We then quasi-resonantly read out emitters E1 and E2, which give us $\nu_1(F_1(-200\text{V}))$ and $\nu_2(F_2(-200\text{V}))$. We proceed by exposing emitter E2 to above-band light

while biased at +200 V. After turning off the laser and the bias, we quasi-resonantly measure the emission of emitters E1 and E2, which gives us the $\nu_1(F_1(-200\text{V}) + \delta F_{1,2}(+200\text{V}))$ and $\nu_2(F_2(-200\text{V}) + \delta F_{2,2}(+200V))$ spectra.

Based on the location of the measured emission frequencies along parabolic fits such as the one shown in Fig. 4b of the main text, we extract the corresponding ambient electric field at the quantum dot due to carrier redistribution and hence the values of δF_{ij}.

Supplementary References

[1] Meitl, M. A. *et al.* Transfer printing by kinetic control of adhesion to an elastomeric stamp. *Nat. Mater.* **5**, 33–38 (2006).

[2] Kim, T.-H. *et al.* Full-colour quantum dot displays fabricated by transfer printing. *Nat. Photon.* **5**, 176–182 (2011).

[3] Justice, J. *et al.* Wafer-scale integration of group III–V lasers on silicon using transfer printing of epitaxial layers. *Nat. Photon.* **6**, 610–614 (2012).

[4] Katsumi, R., Ota, Y., Kakuda, M., Iwamoto, S. & Arakawa, Y. Transfer-printed single-photon sources coupled to wire waveguides. *Optica* **5**, 691–694 (2018).

[5] Katsumi, R. *et al.* Quantum-dot single-photon source on a CMOS silicon photonic chip integrated using transfer printing. *APL Photonics* **4**, 036105 (2019).

[6] Katsumi, R. *et al.* In situ wavelength tuning of quantum-dot single-photon sources integrated on a CMOS-processed silicon waveguide. *Appl. Phys. Lett.* **116**, 041103 (2020).

[7] Katsumi, R. *et al.* CMOS-compatible integration of telecom band InAs/InP quantum-dot single-photon sources on a Si chip using transfer printing. *Appl. Phys. Express* **16**, 012004 (2023).

[8] Bower, C. A. *et al.* Printing microleds and microics for next generation displays (2018). URL `https://www.xdisplay.com/wp-content/uploads/2020/05/2018_08_30_IMID-updated.pdf`.

[9] Timurdogan, E. *et al.* Apsuny process design kit (pdkv3.0): O, c and l band silicon photonics component libraries on 300mm wafers. In *Optical Fiber Communication Conference (OFC) 2019*, Tu2A.1 (Optica Publishing Group, 2019).

[10] Kim, J.-H. *et al.* Hybrid integration of solid-state quantum emitters on a silicon photonic chip. *Nano Lett.* **17**, 7394–7400 (2017).

[11] Aghaeimeibodi, S., Lee, C.-M., Buyukkaya, M. A., Richardson, C. J. & Waks, E. Large Stark tuning of InAs/InP quantum dots. *Appl. Phys. Lett.* **114**, 071105 (2019).

[12] Lee, C.-M. *et al.* Bright telecom-wavelength single photons based on a tapered nanobeam. *Nano Lett.* **21**, 323–329 (2020).

[13] Oskooi, A. F. *et al.* Meep: A flexible free-software package for electromagnetic simulations by the FDTD method. *Comput. Phys. Commun.* **181**, 687–702 (2010).

[14] Uppu, R. *et al.* Scalable integrated single-photon source. *Sci. Adv.* **6**, eabc8268 (2020).

[15] Davanco, M. *et al.* Heterogeneous integration for on-chip quantum photonic circuits with single quantum dot devices. *Nat. Commun.* **8**, 889 (2017).

[16] Kako, S. *et al.* A gallium nitride single-photon source operating at 200 K. *Nat. Mater.* **5**, 887–892 (2006).

[17] Schwartz, M. *et al.* Fully on-chip single-photon Hanbury-Brown and Twiss experiment on a monolithic semiconductor–superconductor platform. *Nano Lett.* **18**, 6892–6897 (2018).

[18] González-Ruiz, E. M., Das, S. K., Lodahl, P. & Sørensen, A. S. Violation of Bell's inequality with quantum-dot single-photon sources. *Phys. Rev. A* **106**, 012222 (2022).

[19] Grim, J. Q. *et al.* Scalable in operando strain tuning in nanophotonic waveguides enabling three-quantum-dot superradiance. *Nat. Mater.* **18**, 963–969 (2019).

[20] Fry, P. W. *et al.* Inverted electron-hole alignment in InAs-GaAs self-assembled quantum dots. *Phys. Rev. Lett.* **84**, 733–736 (2000).

[21] Ostapenko, I. A. *et al.* Large internal dipole moment in InGaN/GaN quantum dots. *Appl. Phys. Lett.* **97**, 063103 (2010).

[22] Miller, D. A. B. *et al.* Band-edge electroabsorption in quantum well structures: The quantum-confined Stark effect. *Phys. Rev. Lett.* **53**, 2173–2176 (1984).

[23] Miller, D. A. B. *et al.* Electric field dependence of optical absorption near the band gap of quantum-well structures. *Phys. Rev. B* **32**, 1043–1060 (1985).
